# Commentary: Nicotinic Acetylcholine Receptor α9 and α10 Subunits Are Expressed in the Brain of Mice

**DOI:** 10.3389/fncel.2018.00104

**Published:** 2018-05-01

**Authors:** Barbara J. Morley, Paul Whiteaker, Ana B. Elgoyhen

**Affiliations:** ^1^Boys Town National Research Hospital, Omaha, NE, United States; ^2^Division of Neurobiology, Barrow Neurological Institute, Phoenix, AZ, United States; ^3^CONICET, Instituto de Investigaciones en Ingeniería Genética y Biología Molecular Dr. Héctor N. Torres (INGEBI), Buenos Aires, Argentina; ^4^Facultad de Medicinia, Instiuto de Farmaologia, Universidad de Buenos Aires, Buenos Aires, Argentina

**Keywords:** Alpha9, alpha10, nicotinic acetylcholine receptors (nAChR), brain, mouse, antibodies, immunohistochemistry (IHC)

In a recent paper published in *Frontiers in Cellular Neuroscience*, Lykhmus et al. (2017) propose that the α9 and α10 nicotinic acetylcholine receptor (nAChR) subunits are present in the brain and may be assembled with the α7 subunit. Their conclusions are based on RT-PCR amplification and antibody labeling. These findings are not supported by a vast accumulation of data reported over the last 22-plus years. Therefore, if correct, their results could result in re-interpretation of a large number of solid and reproducible published studies. A careful examination of the data is warranted.

The α9 subunit was first identified in a rat olfactory epithelium cDNA library (Elgoyhen et al., 1994). *In situ* hybridization studies localized α9 to rat cochlear (Elgoyhen et al., 1994; Morley et al., 1998) and vestibular hair cells (Hiel et al., 1996; Simmons and Morley, 2011), the nasal epithelium, the pars tuberalis of the pituitary (Elgoyhen et al., 1994), and bone marrow (Luo et al., 1998), but not in rat adult and embryonic brain (Elgoyhen et al., 1994). It should be noted that in their 1994 publication Elgoyhen and co-workers only showed a minor subset of their *in situ* hybridization results, since signal was not detected in embryonic and adult brain sections. However, it was stated in their manuscript that *in situ* hybridizations performed over 20 μm coronal sections that were collected every 200 μm through the entire adult brain under different experimental conditions and exposure times, to optimize hybridization conditions, repeatedly provided no evidence of α9 expression in the central nervous system. In these brain coronal sections, α9 signal was only observed in the ventral part of the median eminence, which corresponds to the pars tuberalis of the pituitary (Elgoyhen et al., 1994). In addition, no α9 cDNA clones were obtained from several rat brain cDNA libraries, including total brain forebrain, astrocytes, superior colliculus, and hippocampus, by hybridization screening with a radiolabeled rat α9 DNA fragment (Elgoyhen, unpublished observations). These libraries have been successfully used over and over to clone neuronal nicotinic cholinergic receptor subunits and AMPA and kainate glutamate receptor subunits in the Heinemann laboratory. The absence of α9 in brain by RT-PCR has also been reported in rat (Morley et al., 1998) and trout (Drescher et al., 2004). Moreover, updated RefSeq data published in September, 2017^1^ and *in situ* hybridization data published in the Allen Brain Atlas^2^ confirm these findings. Taken together these results indicate that the α9 gene is not transcribed in the brain.

Lykhmus et al. acknowledge that their data is inconsistent with those findings. They report that they amplified α9 and α10 transcripts from brain samples. Although the resulting products were sequenced, there was no positive control and no Ct-value reported. Inclusion of a positive control, such as the cochlea, vestibule, or pituitary, would have provided a reference point. There was also no negative control, since all brain regions used in their PCR reactions showed positive results.The investigators explained their findings by stating that levels of mRNA below the level detected by RefSeq are often unrelated to protein levels. Although low levels of transcript can produce measureable protein levels, such wide discrepancy is rare, and requires further substantiation. Lack of α9 protein in brain has been reported by Zuo et al. (1999). In that paper, a GFP reporter α9 transgenic mouse was generated that had ~8 times greater abundance of α9 protein compared to endogenous protein in wild type mice. Using antibodies against GFP, Zuo et al. (1999) visualized and localized α9 protein in the same regions where others reported mRNA using radiolabeled probe *in situ* hybridization. However, they found no α9 protein in brain. Moreover, Luebke and Foster (2002) reported no α9 protein in brain using Western blot, but did find robust α9 protein expression in the positive controls (cochlea and pituitary). Therefore, contrary to Lykhmus et al., these data also indicate the lack of α9 protein expression in the brain.

In addition to RT-PCR, the investigators attempted to localize α9 receptors in tissue slices with biotinylated α-conotoxin PeIA (α-CtxPeIA) and biotinylated non-commercial antibodies. The tissue used was fixed by immersion in 4% formaldehyde for 48 h. The results and interpretation of the data are problematic. The novel biotinylated α-CtxPeIA derivative was not characterized or validated. Generously assuming that biotinylation did not alter the affinity of α-CtxPeIA and that heavy fixation did not interfere with ligand binding, the ligand would label receptor sites other than α9 subunits. In particular, the biotinylated α-CtxPeIA concentration used by Lykhmus et al. was 25 nM. The IC50 of α-CtxPeIA at α3β2-nAChR is 9.7 nM, 11.1 nM at α6/3β2β3 nAChR (Hone et al., 2012) and 20–30 nM at α9α10 nAChR (McIntosh et al., 2005; Hone et al., 2012). Despite this, Lykhmus et al. did not include controls to eliminate the possible labeling of other nAChR subtypes.

Moreover, the kinetics of relief from α-CtxPeIA blockade of α9α10 reported by McIntosh et al. (2005) indicates that more than 50% of block is relieved following 3 min of washing and total recovery of function is seen within 12–15 min. In Lykhmus et al., they reported that sections were washed after application of biotinylated α-CtxPeIA for 3 × 20 min. Since the half-life for dissociation from α9α10 is <3 min, this corresponds to >20 half-lives. Thus, the wash time exceeded the half-life of dissociation of specific ligand binding by >20 times. Less than one part in a million (1:2^20^) of the original binding would remain. Therefore, the labeling by α-CtxPeIA cannot be specific. The authors report that the distribution of α-CtxPeIA is very similar to that of α9 antibody labeling in the CA3 region of the hippocampus. This fact casts severe doubt on the accuracy of the immunohistochemical data as well.

New specific antibodies to any nAChR would be welcome, since application of antibodies specific to receptor subunits is a powerful methodology. However, antibodies to nAChRs are notorious for being non-specific when used in immunohistochemistry on fixed tissues (e.g., Jones and Wonnacott, 2005; Moser et al., 2007; Garg and Loring, 2017). In Lykhmus et al., the investigators utilized non-commercial antibodies produced in rabbit against α7, α9, and the α10 subunit peptides on sections from brain tissue (fixed by immersion in 4% formaldehyde for 48 h, as used for the α-CtxPeIA experiments). It has become standard protocol to remove blood from brain by perfusion with saline or buffer and to fix the tissue for short time periods. This increases specificity and sensitivity, and retains intact morphology, but was omitted by Lykhmus et al. This step is particularly important because nAChR subunits (including α9 and α10)-expressing immune cells (e.g., Peng et al., 2004; Hao et al., 2011; Koval et al., 2011; Simard et al., 2013; Jiang et al., 2016; St-Pierre et al., 2016; Liu et al., 2017) and hematopoietic stem cells (Zablotni et al., 2015) circulate in the blood found in brain. The micrographs presented in the paper suggest regions of poor fixation (see Figure 4F). The antibodies were biotinylated and this may affect the affinity of some antibodies. The α9 antibody was used in a dilution 1:50 with 1% BSA as the only blocker and no antigen absorption control was reported. Moreover, the data would be more convincing if controls for non-specific labeling (as just outlined) had been used and if positive controls had been provided. The discrete expression of α9 and α10 in hair cells in the cochlea is well-documented, making it highly practical to determine if the antibodies specifically label receptors on hair cells. The investigators report some regional distribution of α9, α10, and α7 subunits in wild type mice. Since this is the first report of α9 and α10 in brain (all previous studies have shown no expression) there is no other antibody data with which to compare their study. However, α7 has been extensively studied in brain using α-bungarotoxin binding and *in situ* hybridization. The micrographs presented by Lykhmus et al. are of small brain areas. Therefore, it is difficult to compare their data with previously published studies of either the cellular or regional distributions of α7 transcription or translation.

An ELISA assay was used to confirm the immunohistochemical data. The results are difficult to interpret. The data reported in Figure 1 indicates that the levels of α7 and α9 are similar, although the authors acknowledge that the α9 and α10-positive cells in their preparations were rare. It is well-known that α7 is very highly expressed in brain while the density of α9 is below the level of detection by RefSeq. In Figure 2 it was reported that they captured nAChR subunits from wild type mouse brain using a α7 antiibody (α7 1–208) that recognizes the whole extracellular domain and then quantified subunit protein expression using antibodies purported to be specific to α3, α4, α5, α7, α9, and α10. Using this technique, they found that the quantity of α4 and α7 were equal and both of much greater magnitude than β2. Moreover, the quantities of α9 and α10 were reported to be almost as high as β2. These data contradict a large body of literature established with several different techniques that β2 and α4 are the most prevalent subunits in the brain and are more abundant than α7 (e.g., Marks et al., 2010).

Although they report regional differences in density using RT-PCR, the data presented (Figure 3) show only slight quantitative differences among the sampled brain areas. Further, relative regional expression densities of mouse-brain α7-nAChR measured by ELISA in Figure 3 do not fit well with established α-bungarotoxin binding distributions. Please note that α-bungarotoxin binding sites in the CNS have been validated to correspond to α7 (and not the α-bungarotoxin sensitive α9)-nAChR, both by use of nAChR α7 subunit-null mutant mice as negative controls (Orr-Urtreger et al., 1997), and by comparison of their distribution to that of an α-conotoxin derivative (α-CtxArIB[V11L,V16D]) demonstrated to have extreme selectivity for α7-nAChR (Whiteaker et al., 2007). The ELISA results, for example, show that hippocampus expresses a high density of α7 subunits, which does fit well with present knowledge of how this subtype is distributed based on autoradiography (Whiteaker et al., 1999). But they show similar densities in the frontal cortex (which has a modest density expression of α7-nAChR) and “thalamus and putamen” (thalamus has a very low α7-nAChR density, caudate/putamen has an intermediate density) (e.g., in dissected regions of mouse brain; Whiteaker et al., 1999). While the ELISA and RT-PCR results reported by Lykhmus et al. may differ marginally from detailed autoradiography reports because the delineation of regions is less precise in dissected samples, there appears to be a low correlation between the levels of expression indicated by ELISA results (Figure 3A) and the band intensities shown in the accompanying RT-PCR panel in their own data (Figure 3B).

The Lykhmus et al. data are also not consistent with what is known from studies of knockin (ki) mice. For example, the authors show α9- and α10-positive cells in ordered structures or zones, such as the cerebellum. They suggest that α9- and α10-containing nAChRs may be involved in regulating motor coordination. Hypersensitive knockin mice bearing mutations at the highly conserved Leu 9′ residue present at the channel pore region have been generated for several nAChRs (Lester et al., 2003). The replacement of Leu 9′ by a polar amino acid renders receptors that are hypersensitive to agonists, shift the activation/desensitization ratio toward activation, exhibit spontaneous channel openings and decreased desenstitization rates (Revah et al., 1991; Filatov and White, 1995; Labarca et al., 1995; Plazas et al., 2005). Homozygous L9′T α7 or L9′S α4 knockin mice are neonatal lethal (Orr-Urtreger et al., 2000; Labarca et al., 2001). Neuronal cell death is observed in brain regions expressing these receptors, most likely due to Ca^2+^ excitotoxicity and apoptosis. The α9 L9′T hypersensitive mutant mouse, in contrast, is not neonatal lethal and does not show an overt nervous system phenotype (Taranda et al., 2009). If α9 protein was expressed throughout the brain, as described by Lykhmus et al. and having α9 and α9α10 high calcium permeability (Elgoyhen et al., 2000; Katz et al., 2000; Weisstaub et al., 2002; Elgoyhen and Katz, 2012), overt neuronal cell death and centrally-mediated phenotypes, such as locomotion problems would be expected. The absence of this effect provides further (in this case circumstantial) evidence that α9 nAChR expression is not widespread in the brain.

Finally, Lykhmus et al. suggest that the α9 and 10 nAChRs may be expressed in mitochondria, even though they state that the antibodies stained mainly neurons and hypertrophied astrocytes. Co-labeling with antibodies specific to synapses, neurons, or mitochondria was not investigated.

Given all the above considerations, the staining with α9 antibodies in wild-type mice and lack of staining in α9 knockouts is intriguing. One wonders if experiments in both genotypes were performed side by side at the same time and with exactly identical experimental conditions. Taken together, although puzzling, the results need to be replicated using other techniques with more controls for non-specificity, and positive controls to show that the antibodies and probes are recognizing known structures across the brain and within the auditory system. Co-labeling with validated antibodies to specific organelles is necessary to make any conclusions regarding the localization of α9 and α10 within the brain. Speculations regarding a brain function for α9 and α10 nAChRs at this time are unwarranted.

## Author contributions

All authors listed have made equal substantial, direct, and intellectual contributions to the work, and approved it for publication.

### Conflict of interest statement

The authors declare that the research was conducted in the absence of any commercial or financial relationships that could be construed as a potential conflict of interest.

## Footnotes

^1^https://www.ncbi.nlm.nih.gov/gene/231252.

^2^© 2015 Allen Institute for Brain Science. Allen Brain Atlas API. Available from: brain-map.org/api/index.htm.
